# A lifelong bronchopleural fistula after sleeve lobectomy and salvage pneumonectomy

**DOI:** 10.1016/j.xjtc.2025.03.007

**Published:** 2025-03-19

**Authors:** Yihong Ni, Zihan Wang, Yu Han, Kunsong Su, Fei Xiao, Chaoyang Liang

**Affiliations:** a Department of Surgery, Chinese Academy of Medical Sciences and Peking Union Medical College, Beijing, China; b Department of Thoracic Surgery, China-Japan Friendship Hospital, Beijing, China

**Figure:**
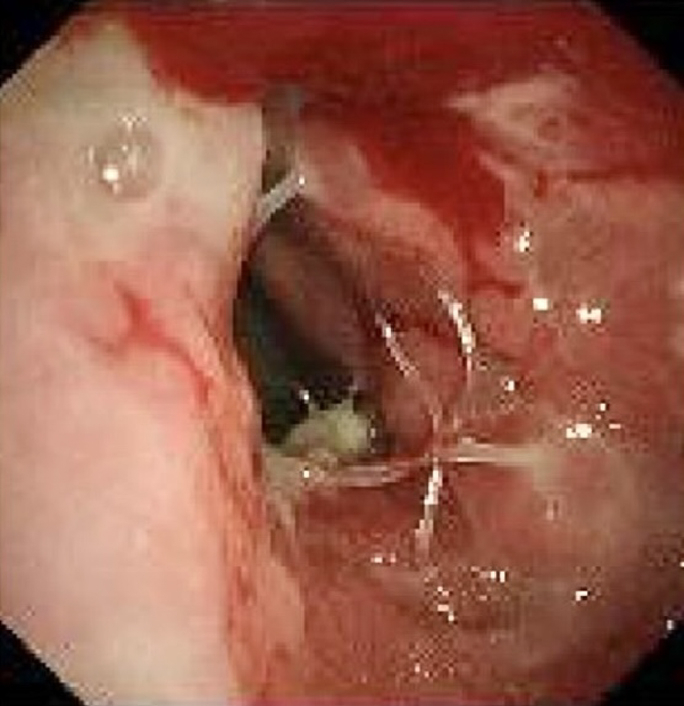
The fistula that remains open permanently after the removal of a tracheal stent.

**Central Message**

For bronchopleural fistulas that are challenging to close, allowing the patient to live with an open fistula may be a possible approach.

Bronchopleural fistula (BPF) is a life-threatening postlung surgery complication. Standard management focuses on fistula repair and closure.^1^ We present a case of a patient with lung squamous cell carcinoma who developed a BPF after sleeve resection and subsequent salvage pneumonectomy. After surgical and interventional treatments failed, we helped the patient achieve gradual stabilization of his general condition, and eventually, self-drainage of the BPF was achieved. Conversion to a similarly limited tracheal diverticulum led to long-term survival and satisfactory quality of life for the patient.

Informed consent for the publication of this case report was obtained from the patient; institutional review board approval was not required.

## Case Report

**Figure 1:**
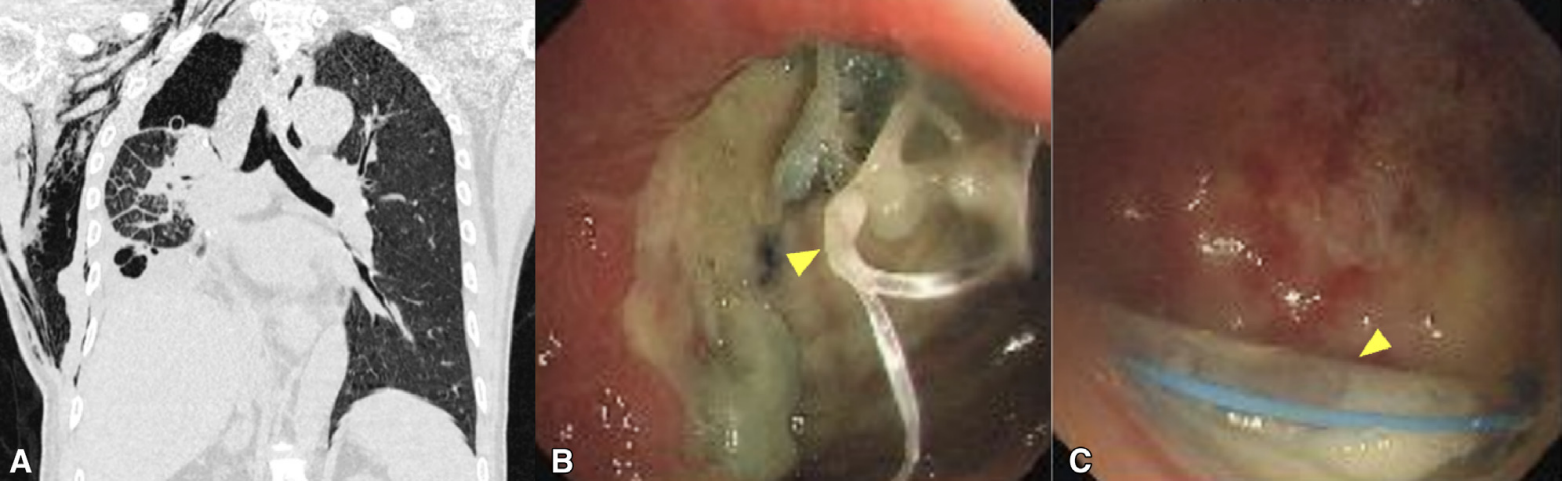
A, Computed tomography image showing a right-sided hydropneumothorax and subcutaneous emphysema. B, A fistula at the right main bronchial anastomosis with a broken suture (*arrow*). C, The right thoracic cavity and drainage tube (*arrow*) are visible through the fistula.

The patient was a 52-year-old man diagnosed with T2 N0 M0 squamous cell carcinoma in the right lower lobe that extended to the right middle bronchus. He underwent a video-assisted right middle and lower sleeve lobectomy. Bedside bronchoscopy on postoperative day (POD) 1 showed an intact anastomosis without evidence of a fistula. Minor air leak led to prolonged chest tube drainage until POD 8. After tube removal, the patient developed fever, cough, and chest tightness. Because computed tomography (CT) revealed a right-sided hydropneumothorax, closed drainage of the thoracic cavity and broad-spectrum antibiotics were initiated. On POD 22, CT scans revealed progressive hydropneumothorax (Figure 1, *A*), and bronchoscopy revealed a fistula with a broken suture at the anastomosis of the right main bronchus (Figure 1, *B*). The chest tube was visible through the fistula (Figure 1, *C*).

**Figure 2:**
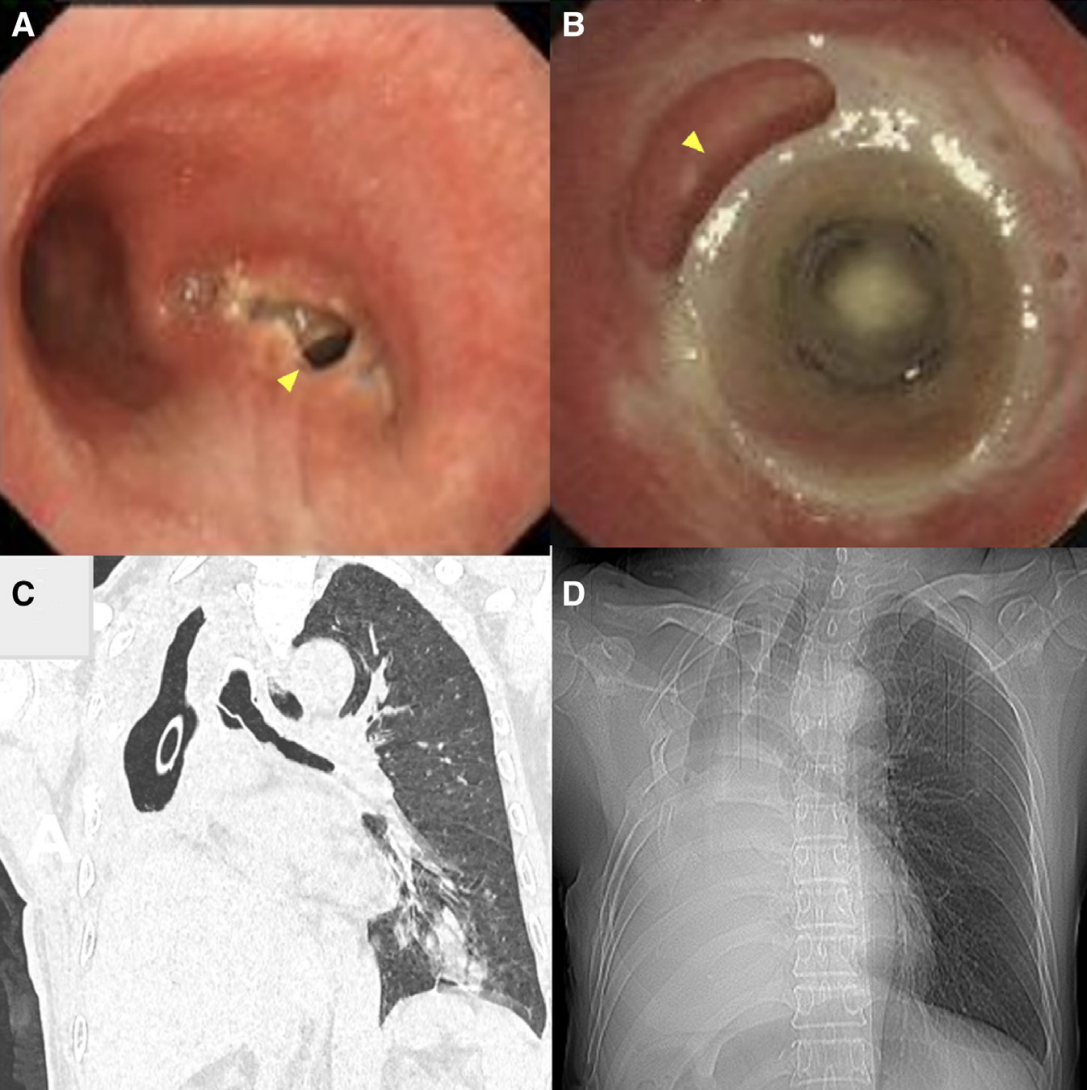
A, A fistula (*arrow*) formed at the stump of the right main bronchus. B, The gap (*arrow*) between the stent and the bronchus. C, Inflammation of the left lung and a hydropneumothorax in the right lung. D, A tracheal diverticulum structure formed by a bronchopleural fistula.

A salvage pneumonectomy was then performed. The remaining right upper lobe was resected and the bronchial stump was sutured and wrapped with an intercostal muscle flap. On postreoperation day 8, bronchoscopy revealed a new BPF in the right main bronchial stump (Figure 2, *A*). To address this new fistula, a modified silicone stent was placed in the trachea and left main bronchus to cover the fistula. The intervention markedly improved the patient's condition and resolved the consistent air leak.

One month later, the patient developed constant cough and a tremendous amount of sputum. Bronchoscopy revealed the proximal end of the stent did not completely adhere to the bronchus to cover the fistula (Figure 2, *B*). CT demonstrated a localized infection of the left lower lobe (Figure 2, *C*). At this point, because his right thoracic cavity was severely compressed according to the images, we hypothesized that the remaining fistula might not be life-threatening, and the small thoracic cavity could shift to a structure-like tracheal diverticulum permanently. With the patient's consent, the stent was removed. Antibiotics were ceased and the drainage was gradually clamped after infection was cleared by monitoring the drainage fluid, blood markers, and images. Three months following the removal of the chest tube, the patient resolved with an asymptomatic open bronchopleural fistula (Figure 2, *D*, and Video 1).

## Discussion

Large fistulas can be surgically repaired when the patient's general condition permits surgery. Tissues such as intercostal muscle flaps, pericardium, and fat can be used to wrap fistulas and reinforce bronchial stumps to help prevent BPF.^2^ In this case, pneumonectomy and intercostal muscle flap were chosen to resolve the first BPF, which failed. For empyema secondary to BPF, open window thoracostomy, such as the Eloesser flap, is a classic but invasive option.^3^ Due to its significant trauma and limited patient acceptance, we chose double chest tube drainage for effective infection control. For smaller BPFs (<8 mm), interventional treatments, including modified silicone stent placement, offer effective and safe alternatives.^4^ The stent provided temporary relief but ultimately did not resolve the issue. After conservative, surgical, and interventional treatments failed, we attempted to keep the fistula open by converting the right thoracic cavity into a tracheal diverticulum structure to help the patient achieve a satisfactory quality of life.

## Conclusions

This case provides a new way of handling BPFs. Instead of eliminating all fistulas, perhaps it is practical to live with them.

## Conflict of Interest Statement

The authors reported no conflicts of interest.

The *Journal* policy requires editors and reviewers to disclose conflicts of interest and to decline handling or reviewing manuscripts for which they may have a conflict of interest. The editors and reviewers of this article have no conflicts of interest.

## Supplementary Data

Video 1 Chest computed tomography imaging. Video available at: https://www.jtcvs.org/article/S2666-2507(25)00092-6/fulltext.

## References

[1] Puskas J.D., Mathisen D.J., Grillo H.C., Wain J.C., Wright C.D., Moncure A.C. (1995). Treatment strategies for bronchopleural fistula. J Thorac Cardiovasc Surg.

[2] Steimer D., Coughlin J.M., Yates E. (2024). Empiric flap coverage for the pneumonectomy stump: how protective is it? A single-institution cohort study. J Thorac Cardiovasc Surg.

[3] Iioka S., Sawamura K., Mori T. (1985). Surgical treatment of chronic empyema. A new one-stage operation. J Thorac Cardiovasc Surg.

[4] Jin L., Li Y. (2023). Bronchoscopic interventions for bronchopleural fistulas. Ther Adv Respir Dis.

