# Supplementary figures and images for: A lifelong bronchopleural fistula after sleeve lobectomy and salvage pneumonectomy
Source: JTCVS Tech. 2025 Mar 19;31:182–4. doi: 10.1016/j.xjtc.2025.03.007 (PMC12237853; doi:10.1016/j.xjtc.2025.03.007)

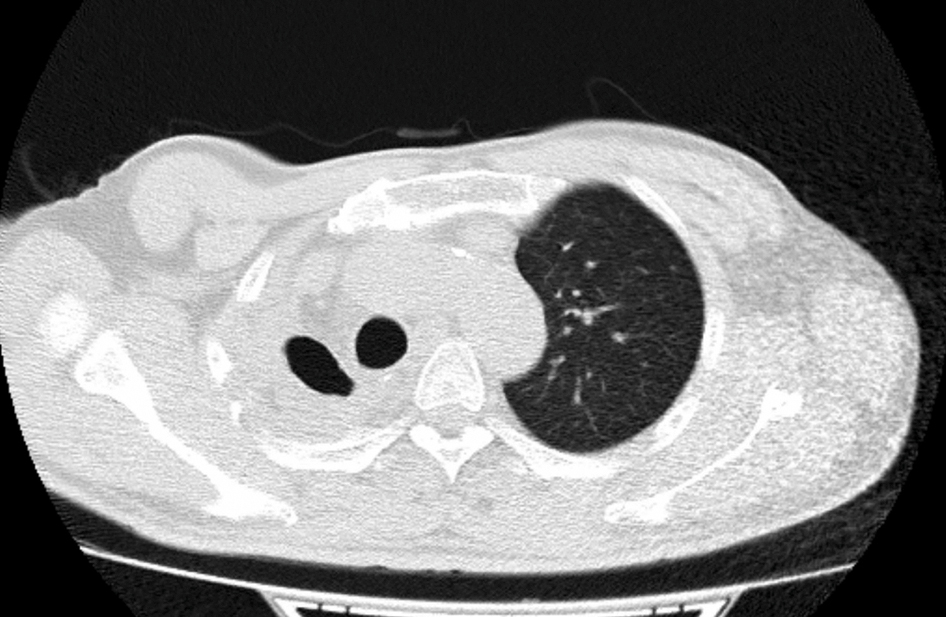

Supplement: Video 1 — Chest computed tomography imaging. Video available at: https://www.jtcvs.org/article/S2666-2507(25)00092-6/fulltext. [file fx2.jpg]
